# Maternal Warning Signs Education During Home Visiting: Results from a Formative Evaluation in Maryland

**DOI:** 10.1089/whr.2022.0027

**Published:** 2022-07-11

**Authors:** Jennifer A. Callaghan-Koru, Inaya Wahid, Shari M. Lawson, Kelly M. Bower, Colleen S. Wilburn, Andreea A. Creanga

**Affiliations:** ^1^ Department of Sociology, Anthropology, and Public Health, University of Maryland, Baltimore County, Baltimore, Maryland, USA.; ^2^ Department of Gynecology and Obstetrics, Johns Hopkins University School of Medicine, Baltimore, Maryland, USA.; ^3^ School of Nursing, Johns Hopkins University, Baltimore, Maryland, USA.; ^4^ Maternal and Child Health Bureau, Maryland Department of Health, Baltimore, Maryland, USA.; ^5^ Department of International Health, Johns Hopkins Bloomberg School of Public Health, Baltimore, Maryland, USA.

**Keywords:** health disparities, health education, home visiting, maternal health

## Abstract

**Background:**

Maternal mortality rate reviews have identified the need for improved patient education regarding the warning signs of maternal complications to reduce preventable deaths. Maternal and child home visiting programs have the potential to deliver this education in communities.

**Aims:**

This study sought to evaluate the baseline provision of warning signs education among home visiting programs in Maryland and to assess the acceptability of and preferences for warning signs education materials among program staff.

**Materials and Methods:**

This sequential exploratory, mixed-methods study included qualitative interviews and focus group discussions followed by a web-based survey of all home visiting programs providing early postpartum visits in Maryland.

**Results:**

Twenty-five home visiting program staff took part in qualitative data collection, and survey responses were submitted by a manager from 40 of 58 eligible home visiting programs (69% response rate). All survey respondents agreed that home visiting programs should provide warning signs education and more than 80% of programs provided some warning signs education during pregnancy and the postpartum period. Printed pamphlets were provided by 68% of programs for pregnancy complications and 43% for postpartum complications. Only 33% of respondents were satisfied with their existing warnings signs education materials; 98% were interested in new illustrated pamphlets and 88% were interested in education videos. Qualitative participants considered pamphlets with simple designs, limited text, and visuals, as the most accessible for home visiting clients.

**Conclusions:**

There are opportunities to strengthen and expand warning signs education in Maryland through home visiting programs using new printed and video education materials.

## Background

The status of maternal health in the United States has been characterized as a crisis. Maternal mortality and morbidity rates in the United States are higher than in other high-income countries.^1^ The maternal mortality rate, as reported by the Centers for Disease Control and Prevention (CDC), has steadily risen from <10 deaths per 100,000 live births before 1990 to >15 deaths per 100,000 for every year since 2010.^2^ Although some analyses attribute part of the increases in maternal mortality rate to changes in measurement,^1,3,4^ the rates of severe maternal morbidity during delivery hospitalizations also rose from <50 per 10,000 delivery hospitalizations in 1993 to almost 150 per 10,000 delivery hospitalizations in 2014.^5^

The US maternal health crisis is particularly acute for non-Hispanic black women, who have 2.5 times the risk of pregnancy-related deaths and 1.7 times the risk of severe maternal morbidity, when compared with non-Hispanic white women.^4,6^ State-based maternal mortality review committees have determined that two-thirds of maternal deaths were preventable.^7^ Causes of pregnancy-related deaths that can be prevented include infection (12.5%), obstetric hemorrhage (11%), cardiomyopathy (11%), and hypertensive disorders of pregnancy (6.9%).^2^

Reviews of maternal deaths indicate that lack of knowledge about the signs of maternal complications among patients and families is a contributing factor in many deaths.^8^ Despite the importance of timely recognition and care seeking for signs of maternal complications, there have been few studies in the United States assessing pregnant and postpartum patients' knowledge of the signs of maternal complications and the education health care providers deliver to patients on this topic. A 2017 survey of postpartum nurses found that most nurses spent less than ten minutes educating patients about warning signs before discharge from delivery, and there was substantial variability in which complications nurses discussed with patients.^9^ A 2016 survey of women who delivered at an academic medical center asked participants to identify warning signs of serious postpartum complications.^10^ Among the nine warning signs assessed in the study, five were not known to the majority of respondents.^10^

The CDC has recommended improving patient education on maternal warning signs to promote timely recognition of, and care seeking for, serious maternal complications.^8^ Recently, organizations such as the Association for Women's Health, Obstetric and Neonatal Nursing (AWHONN) and the Council on Patient Safety in Women's Health Care (hereafter, the Council) have developed standardized warning signs education materials.^9,11,12^ AWHONN's postpartum warning signs pamphlet, introduced in 2016, was designed for postpartum discharge education, and includes an implementation tool kit for hospitals.^13^ The Council's education materials cover both pregnancy and postpartum complications and were introduced in 2020.^12^

While implementation of standardized warning signs education has thus far focused on discharge from the delivery hospitalization, there are limitations with this approach. First, approximately one-third of maternal deaths occur during pregnancy, and another one-third occur between 7 days and 1 year postpartum,^8^ suggesting the need for ongoing warning signs education across the perinatal care continuum. Second, the education provided to postpartum patients before discharge may be difficult to retain due to pain, stress, or mental overload experienced during their delivery hospitalization.^14^

Maternal and child home visiting programs present a potential opportunity to provide at-risk patients with timely antenatal and postpartum warning signs education in the context of a long-term, trusted relationship. Federally-funded home visiting programs, such as the Maternal, Infant and Early Childhood Home Visiting (MIECHV) Program and the Healthy Start program, serve low-income families in communities with poor health outcomes.^15^ In Maryland, more than 60 different home visiting programs operate throughout the state.^16^ While the focus of home visiting programs has traditionally been on the child's health and well-being,^15^ most home visiting models include prenatal visits and frequent visits in the early postnatal period. These contacts with women and their families are a potential delivery point for timely education and screenings for maternal health.

Through a Maternal Health Innovation Program grant from the Health Resources and Services Administration,^17^ the statewide Maryland Mom (MDMOM) Program undertook an initiative to adapt and scale up maternal warning signs education for home visiting programs. This article reports the results of a mixed-methods formative evaluation aiming to (1) describe the baseline provision of warning signs education among home visiting programs in Maryland; and (2) assess the acceptability of and preferences for warning signs education materials among home visiting program staff.

## Materials and Methods

Following a sequential-exploratory design,^18^ this study collected qualitative and quantitative data from home visiting program staff between June and October 2020. The study was approved by the Institutional Review Board of the University of Maryland, Baltimore County (Protocol No. Y20JCK21197). All participants provided informed consent and were offered a $30 gift card. Data collection and analysis methods are described in detail by procedure.

### In-depth interviews and focus group discussions with program staff

The Maryland Department of Health circulated an invitation to participate in qualitative data collection to managers from all home visiting programs in Maryland and asked program managers to distribute the invitation to their staff. The invitation e-mail provided the link to an online form where individuals could volunteer to participate by providing their contact information and availability. Due to the coronavirus pandemic, all interviews and focus group discussions (FGDs) took place virtually, through WebEx, between June and July of 2020. The study team scheduled FGDs among volunteers with equivalent program roles (*e.g.*, home visitor, manager) who indicated the same availability. Individual interviews were scheduled for all other volunteers. Before each interview or discussion, participants returned fillable PDF consent forms and demographic questionnaires by e-mail.

Interviews and focus groups were led by one of three facilitators, all with graduate-level training in qualitative research methods, and included one notetaker. Semistructured guides included questions that addressed the participants' role in home visiting, maternal health concerns expressed by clients, coordination between home visiting programs and maternity care providers, the types of education provided by the program on maternal health and warning signs, and how health education in home visiting had changed as a result of COVID-19.

Participants were also asked to provide their perceptions of the feasibility and acceptability of including warning signs education and blood pressure screenings during home visits. Through a visual elicitation exercise,^19^ participants were shown three warning signs education pamphlets from the Council, AWHONN, and SaferCare Texas,^9,12,20^ and were asked to describe the features that they preferred. Interviews and focus groups typically lasted 60 and 90 minutes, respectively, and audio recordings were transcribed for analysis.

Preliminary analysis of the qualitative data was completed following a rapid approach to inform the survey design.^21,22^ Immediately following each interview, the notetaker summarized the participant's responses in a chart with rows for each question on the interview guide. Subsequent qualitative analysis followed an adapted five-step Framework approach.^23^ A preliminary codebook was developed based on the questions from the interview guide. The codebook was applied to six interviews by two research assistants, and uncertainties were discussed in a consensus meeting with the lead author, to refine the operational definitions and add new codes for emerging themes. All transcripts were coded in NVivo 12 Pro using the final consensus codebook. Coded excerpts were summarized in charts by respondent, and memos were developed for each code for data synthesis.

### Survey of home visiting programs

In October 2020, the study team conducted a survey of home visiting programs in Maryland to assess their existing maternal health education and interest in incorporating new education materials on warning signs. Programs that provided home visiting to clients during pregnancy or in the first 4 weeks postpartum were eligible for the survey (see Supplementary Table S1 for further description of program models). The senior managers for each of the 58 organizations with eligible home visiting programs were identified from a list compiled and maintained by the Maryland Department of Health for the biannual legislature-mandated report on home visiting programs.^16^ Program managers received e-mail invitations to complete the survey in Qualtrics, including up to five reminders for those who had not yet completed the survey.

The original survey instrument was revised before distribution to include questions related to health education practices and preferences that emerged from the rapid qualitative data summaries. The final survey included questions that addressed respondent demographics, organizational and program characteristics, and maternal health services for home visiting clients. Respondents' perceptions about and preferences for delivering health education during home visiting were measured by asking respondents to rate their agreement with related statements using a five-point Likert scale. The survey data were imported into Stata 16 for descriptive, univariate analysis.

## Results

Characteristics of the qualitative participants and survey respondents are presented in Table 1. A total of 25 participants were included in qualitative data collection through 15 in-depth interviews and 3 FGDs. Twelve qualitative participants were nonclinical home visitors, four were nurse visitors, and eight were in supervisory roles. Among the survey respondents, 35 held a director or supervisor position, 4 were coordinators, and 1 was a home visitor. Approximately 95% of each sample was female. The qualitative sample had an equal number of white and black/African American participants, while 54% of survey respondents were white and 37% were black/African American.

**Table 1.** Characteristics of Participants

Characteristics	Qualitative participants (*n* = 24^a^)		Survey participants (*n* = 40)	
	No.	%	No.	%
Age				
30 or younger	6	25	2	5
31–40	6	25	9	23
41–50	6	25	15	38
51–60	4	17	9	23
Older than 60	2	8	5	13
Gender				
Female	23	96	38	95
Male	1	4	2	5
Race				
White	11	46	22	55
Black or African American	11	46	14	35
Asian or Pacific Islander	1	4	0	0
More than one race	1	4	0	0
Prefer not to answer	0	0	4	10
Hispanic, Latino, or Spanish origin				
No	21	88	37	93
Yes	3	13	2	5
Prefer not to answer	0	0	1	3
Highest level of education completed				
Some college or associate degree	2	8	1	3
Bachelor's degree	18	75	12	30
Master's degree or higher	4	17	27	68
Position at organization				
Home visitor/educator	12	50	1	3
Nurse visitor	4	17	0	0
Coordinator/administrator	1	4	4	10
Manager/supervisor	5	21	15	38
Director/deputy/assistant director	2	8	20	50
Home visiting program model in use^b^				
Healthy Families America	13	54	—	—
Nurse Family Partnership	3	13	—	—
Parents as Teachers	2	8	—	—
Healthy Start	2	8	—	—
Maternity Partnership	2	8	—	—
Other models^c^	3	13	—	—

^a^ One participant missing.

^b^ Multiple responses allowed.

^c^ Other models include Babies Born Healthy, Early Head Start, Partners for a Healthy Baby.

Among the 58 eligible organizations, 40 (69%) responded to the survey. Twenty-three organizations (58%) were nonprofit and 16 (40%) were local health departments or governments (Table 2). While 31 organizations (78%) only provided one model of home visiting, 9 (23%) provided multiple home visiting programs using different service delivery models. The most common program models were Health Families America (43%), Parents as Teachers (35%), and Early Head Start (28%). The median number of home visitors employed by the responding organizations was 4 (range: 1, 45) and the median annual number of families served was 67 (range: 11, 950). The results from the qualitative and quantitative data analyses are presented together by theme, following an integrated narrative approach.^24^

**Table 2.** Characteristics of Home Visiting Programs Represented in the Survey (*n* = 40)

Characteristics	No.	%
Type of organization		
Nonprofit	23	58
Local health department	10	25
Local/county government	6	15
For-profit	1	3
No. of home visiting program models		
1 Model	31	78
2 Models	4	10
3 Models	3	8
≥4 Models	2	5
Home visiting program models in use^a^		
Healthy Families America	17	43
Parents as Teachers	14	35
Early Head Start	11	28
Healthy Start	3	8
Family Connects Maryland	2	5
Other models^b^	7	18
Funding sources^a^		
State Government	29	73
Federal Government	25	63
Local Government	18	45
Nonprofit or charitable organization	8	20
Median no. of home visiting managers and supervisors (range)	2 (1, 12)	
Median no. of home visitors (range)^c^	4 (1, 45)	
Median no. of families served per year (range)	67 (11, 950)	
Time clients typically receive first prenatal visit^c^		
First trimester	16	41
Second trimester	9	23
Third trimester	5	13
Varies based on when clients are enrolled	9	23
Time clients typically receive first postnatal visit^c^		
Within 7 days after birth	8	21
1–2 Weeks after birth	18	46
3–5 Weeks after birth	5	13
6+ Weeks after birth	4	10
Varies based on when clients are enrolled or on caseload	4	10

^a^ Multiple responses allowed.

^b^ Other models include Attachment Biobehavioral Catch-Up (ABC), Early Care Program, Frog Street Love & Learn, Growing Great Kids, Nurturing Parenting, and Parent Assistance in the Home (PATH).

^c^ One response missing.

### Home visiting programs' role in addressing maternal health concerns

Survey respondents considered home visitors to play an important role in providing health education to clients and addressing their maternal health concerns (Table 3, Section A). Nearly all respondents agreed that home visitors are an important source of health information for clients (98%), and most agreed that clients often ask maternal health questions to home visitors (82%) and for their assistance in finding health information (79%). Although most respondents agreed that home visitors feel comfortable providing general health education to clients (73%), fewer than half agreed that home visitors are comfortable answering health questions from clients (43%).

**Table 3.** Survey Respondents' Agreement with Statements About Health Education in Home Visiting Programs

Statement	No. agree^a^/No. respond	%
**Section A.** Home visiting programs' role in addressing maternal health concerns		
Home visiting programs are an important source of health information for clients.	39/40	98
Clients often have concerns about symptoms that they experience during pregnancy and after delivery.	28/35	80
Clients often ask maternal health questions to home visitors.	28/34	82
Clients often ask home visitors for help with finding health information.	27/34	79
Home visitors feel comfortable providing health education to clients.	27/37	73
Home visitors feel comfortable answering clients' health questions.	16/37	43
**Section B.** Acceptability of warning signs education		
I think home visiting programs should educate clients about the signs of pregnancy and postpartum complications.	40/40	100
I would like our home visitors to receive training on causes and signs of maternal complications.	39/40	98
I am interested in new illustrated pamphlets for educating clients about the signs of maternal complications.	39/40	98
Home visitors think it's important to educate clients about the signs of maternal complications.	33/37	89
Home visitors would like to receive training on the causes and signs of maternal complications.	33/37	89
**Section C.** Preferences for warning signs education materials		
I like the materials that we already have for educating clients about maternal complications.	13/39	33
I am interested in using brief videos to educate clients about the signs of pregnancy and postpartum complications.	35/40	88
Home visitors would like to have a brief video about the signs of maternal complications that they can share with their clients.	29/37	78
Clients would rather learn health information by watching a video than reading a printed pamphlet.	24/35	69
Brief videos are a good way to provide health education to clients during COVID-19.	36/40	90
Home visitors would like to have a website about maternal health complications that they can share with clients.	32/37	86
Clients would like to have access to a comprehensive website about the signs of maternal health complications.	19/35	54
Clients usually keep the printed pamphlets that the home visitors provide to them.	11/35	31

^a^ Includes respondents who rated the statement as “Strongly agree” or “Agree.”

Qualitative research participants also described an important role for home visitors in addressing maternal health concerns. Home visitors reported that first-time mothers, in particular, ask them about what to expect from the delivery process, normal postpartum bleeding, and breastfeeding. Depression and anxiety were the health problems that participants described as the most frequent among clients. Participants also mentioned other maternal health issues that varied in prevalence according to the population served by the program, including gestational diabetes, hypertension, and substance use disorders.

### Existing education on maternal warning signs

Most survey respondents reported that their programs were providing some form of education to clients about the signs of maternal complications during pregnancy (33; 83%) and the postpartum period (36; 90%) (Table 4). Verbal education was most common across programs. Printed pamphlets were provided in 27 programs (68%) for pregnancy complications, but only 17 (43%) for postpartum complications. AWHONN's pamphlet was used by three programs (8%), and other sources of pamphlets included the program's curriculum (*e.g.*, Partners for a Healthy Baby, Growing Great Kids), the March of Dimes, the CDC, and the local health department. Few programs used videos for warning signs education during pregnancy (7; 18%) or postpartum (3; 8%). Only six respondents (15%) indicated that home visitors had received training on the causes and signs of maternal morbidity and mortality.

**Table 4.** Current Provision of Maternal Warning Signs Education by Home Visiting Programs in Maryland (*n* = 40)

Education type	No.	%
Antenatal education		
Currently providing client education on pregnancy complication signs/symptoms	33	83
Type of antenatal education^a^		
Verbal education	33	83
Printed pamphlet	27	68
Video education	7	18
Other type of education	11	28
Postpartum education		
Currently providing client education on postpartum complication signs/symptoms	36	90
Type of postpartum education^a^		
Verbal education	32	80
AWHONN “Save Your Life” pamphlet	3	8
Other printed pamphlet	14	35
Video education	3	8
Other type of education	14	35
Warning signs training		
Home visitors received training on maternal warning signs	6	15

^a^ Multiple responses allowed.

### Acceptability of warning signs education and maternal health screenings

Both the quantitative and qualitative data demonstrated strong support for the inclusion of warning signs education in home visiting services. During qualitative interviews, which initially focused on education for postpartum warning signs, several participants felt it was important to address potential complications during pregnancy as well, so that clients “know how to advocate for [themselves] before, during, and after the pregnancy” (Program Supervisor, Southern Maryland). All survey respondents agreed with the statement, “Home visiting programs should educate clients about the signs of pregnancy and postpartum complications” (40; 100%) (Table 3, Section B).

Nearly all survey respondents agreed that they would like home visitors to receive training on the causes and signs of maternal complications (39; 98%) and that they were interested in new illustrated pamphlets for warning signs education (39; 98%). Slightly fewer respondents agreed that home visitors think it is important to educate clients about maternal warning signs (33; 89%) and that home visitors would like to receive training on the topic (33; 89%).

Qualitative participants were also asked about the acceptability of measuring blood pressure during home visits, a screening that was already provided by nurse home visitors. While some nonclinical home visitors felt comfortable with the idea, the majority expressed concerns about adding blood pressure screenings. These concerns included that it was outside the scope of their training and expectations, that they were not prepared to respond if they identified a client with hypertension, and that medical screenings could confuse clients about their role. One home visitor explained, “I don't want to be [seen as] the nurse because, then they're going to think I know the answers to everything if I pull out a blood pressure cuff” (Home visitor, Eastern Shore).

### Preferences for warning signs education materials and delivery

Among the qualitative participants already providing maternal warning signs education, some liked their existing materials, while others found their materials to be ineffective or inaccessible to clients. The need for new warning signs education materials was more strongly demonstrated by survey respondents—only 13 (33%) agreed with the statement, “I like the materials that we already have for educating clients about maternal complications” (Table 3, Section C).

An important consideration for warning signs education materials expressed by multiple qualitative participants was the accessibility of materials for clients with limited English proficiency or low literacy levels. One home visitor described her challenges with finding effective health education materials for her clients: “A lot of my ladies don't read or they don't read well, so the curriculum is way over their heads” (Home visitor, Eastern Shore). Another home visitor described how she tries to select health educational materials that suit each client: “I have to go according to [each client's] educational level… maybe with the younger clients I might do something with pictures that I can let them see. My older clients, depending on their education, I might do something with written material… or I might just do a combination of both” (Home visitor, Baltimore City).

During the elicitation exercise, qualitative participants identified a variety of strengths and limitations for each of the three warning signs pamphlets that were shown. Features of pamphlets that participants valued included simple designs that were not text heavy, actionable information that distinguishes between emergency signs and concerning signs, and inclusion of mental health concerns. While some participants showed preference for the AWHONN pamphlet, more participants considered the Council's warning signs pamphlet to be the most appropriate for home visiting clients due to its use of visuals and simple text that could be understood by clients with varying literacy levels. Several participants also described the Council's pamphlet as “friendly,” as expressed by one program manager: “This seems to be more friendly, more like, ‘OK we can talk about it together,’ rather than me giving you all this information” (Program manager, Southern Maryland).

### Adapting health education for remote home visiting during the COVID-19 pandemic

While some qualitative participants reported very little change in their interactions with clients during remote visits, more participants reported that clients were not easy to reach and less engaged for phone or video visits. Home visitors described not being able to spend as much time with clients during remote visits and, as a result, they condensed health education and delivered the information with less detail. The majority of programs were still providing hard copy health education materials to clients through periodic no-contact drop-offs to their homes.

One home visitor mentioned in a qualitative interview that texting video links to clients and discussing the video afterward had been a successful adaptation for health education during remote home visiting. Based on this suggestion, the survey asked respondents to rate their interest in using a brief video to educate clients on maternal warning signs (Table 3, Section C). Most respondents agreed or strongly agreed that they would be interested in using videos for maternal warning signs education (88%), and that home visitors would like to have a warning signs video to watch with clients (78%). A smaller majority of respondents agreed that clients would prefer a video to a pamphlet for health education (69%).

## Discussion

The formative evaluation described in this article confirmed the need and interest among home visiting programs in Maryland for improving maternal warning signs education. Managers from a variety of programs and regions of the state overwhelmingly reported that home visitors should provide maternal warning signs education to clients and that clients have a need for this information. The provision of supplemental educational materials is also supported by the policies of many home visiting program models, such as the best practice standards for Healthy Families America—the most commonly used program model in Maryland.^25^ The HFA standards specify that information shared with clients can come from credible sources in addition to curriculum packages and that it should be culturally appropriate for the client population.

While most Maryland programs that responded to the survey were providing some form of maternal warning signs education, less than half had written pamphlets covering postpartum signs, and only one-third of programs were satisfied with their current warning signs education materials. Although warning signs education materials in home visiting have not previously been evaluated, one study assessed hospital discharge materials and published pamphlets for postpartum patients. All materials reviewed in the study were found to be above a fifth grade reading level, having limited compliance with the National Standards for Culturally and Linguistically Appropriate Services.^20,26^

**FIG. 1.**
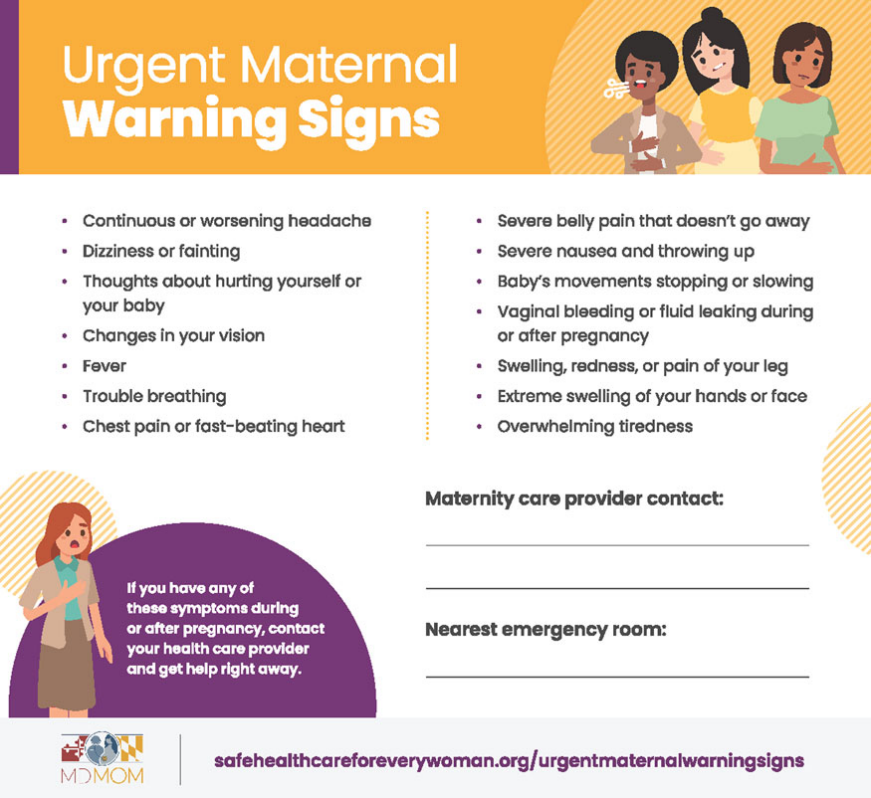
Warning signs education magnet for home visiting programs in Maryland. The Council on Patient Safety in Women's Health Care provided permission to use their list of urgent maternal warning signs.

The qualitative and quantitative feedback from this evaluation also shaped the design of a maternal warning signs tool kit for home visiting programs developed by the MDMOM Program (Box 1). Due to concerns of nonclinical home visitors about conducting blood pressure and other health screenings, the tool kit focuses on health education about maternal warning signs. Qualitative participants' input led to the selection of the Council's maternal warning signs pamphlet and website as the basis for the tool kit. Following the interest expressed by home visiting programs, MDMOM also developed a brief video with obstetricians presenting the warning signs information in English and Spanish (available at: mdmom.org/warningsigns; see Supplementary File S2 for video storyboard). The Council provided permission for the illustrations to be incorporated into the videos as well as a magnet listing the warning signs (see Fig. 1), ensuring consistency across all education materials.

**Box 1.** Components of the Pilot Maternal Warning Signs Education Tool Kit for Home Visiting Programs in Maryland

• Maternal warning signs pamphlet from the Council on Patient Safety in Women's Health Care (including additional translations as needed)
• Brief video presentation of the warning signs by obstetricians in English and Spanish incorporating the Council's graphics
• Refrigerator magnet listing warning signs
• Step-by-step education guide for home visitors with sample language that adapts motivational interviewing techniques and universal health literacy precautions to warning signs education
• Training for home visitors
• Implementation planning guide for program managers

The MDMOM Program also developed a training and education guide for home visitors. The 90-minute training addresses the prevalence and causes of maternal morbidity and mortality; detailed information about the 15 warning signs on the Council's pamphlet; principles of effective health education; and suggestions for how to use the pamphlet, video, and website. The education guide incorporates motivational interviewing techniques, adapted from a published example,^27^ and prompts home visitors to negotiate a plan with clients for saving the information and responding to a warning sign. The tool kit's full educational materials utilize multiple health literacy precautions,^28^ including using graphics, providing concrete signs that are described in plain, nonmedical language, and providing opportunities for repetition of information and reflection. An implementation planning guide provides program managers with guidance on incorporating warning signs education into routine home visits.

The MDMOM Program's maternal warning signs education tool kit allows programs to adapt implementation plans based on the model of services that they provide. Different home visiting program models have varying eligibility guidelines, and individual sites can develop their own more specific criteria based on local priorities^29,30^ (see Supplementary Table S1 for characteristics of program models common in Maryland). Although most program models begin enrollment of clients during pregnancy, many continue to enroll clients after the child is born.^29^ Maternal warning signs education would ideally be delivered to clients during an early prenatal visit with a refresher during the first postpartum home visit. Programs may need to adjust or individualize the timing of warning signs education around the date of enrollment for clients as well as the sequencing and duration of other required activities during home visits.

The tool kit was piloted in five home visiting programs across the state during 2021. Following revisions to address staff and client feedback, the tool kit will be offered to all home visiting programs in Maryland.

This formative research study has several limitations. We were only able to obtain and review copies of the existing warning signs education materials from a small number of programs. Perceptions about the existing materials, as measured in the survey, primarily reflect the opinions of program managers, rather than home visitors. Although managers often make decisions about which supplementary materials their program will provide to clients, they are likely to use the materials less frequently than home visitors. We were also unable to include clients in the data collection for this formative research study. The evaluation protocol for the warning signs education pilot plans to address these gaps through qualitative data collection with clients and home visitors as well as surveys of home visitors.

Attention to improving and standardizing patient education on the signs of maternal complications in the United States is relatively recent. In more resource-limited settings, where patients have difficulty accessing quality care as a result of multiple delays,^31^ patient knowledge of maternal warning signs (often referred to as “danger signs”) has been emphasized and studied for more than two decades.^31–34^ However, reviews of maternal deaths demonstrate that limited knowledge of warnings signs contributes to maternal deaths in the United States as well.^8^ The existing efforts to implement standardized warning signs education in the United States have primarily focused on discharge from the hospital after birth.^35^ Supporting community-based services to provide warning signs education may help increase the reach of these important messages, particularly for underserved patients. The MDMOM Program's tool kit for home visiting programs is one of several similar initiatives underway.

Efforts to improve warning signs knowledge would also benefit from incorporation of knowledge questions into surveys such as the Pregnancy Risk Assessment Monitoring Survey. With reliable measures of patient knowledge, maternal and child health programs will be better able to ensure that they are effectively and equitably meeting the needs of clients and families.

## Conclusions

Providing maternal warning signs education during home visits was considered highly acceptable by managers and staff of maternal and child home visiting programs in Maryland. This assessment identified the need for education that covers both pregnancy and postpartum signs and appropriate education aids, including printed pamphlets and educational videos.

## Supplementary Material

Supplemental data

Supplemental data

## Abbreviations Used

AWHONN: Association for Women's Health, Obstetric and Neonatal Nursing
CDC: Centers for Disease Control and Prevention
FGD: focus group discussion
MDMOM: Maryland Mom
