# Supplementary material for: Maternal Warning Signs Education During Home Visiting: Results from a Formative Evaluation in Maryland
Source: Womens Health Rep (New Rochelle). 2022 Jul 11;3(1):633–42. doi: 10.1089/whr.2022.0027 (PMC9380880; doi:10.1089/whr.2022.0027)
Supplement: Supplemental data [file Suppl_TableS1.docx]

**Supplemental File 1. Maternal warning signs education video storyboard**

| Scene 1 |  | [Intro music.] |
| --- | --- | --- |
| Scene 2 |  | Hello, my name is Shari Lawson, and I’m a general obstetrician/gynecologist at Johns Hopkins Hospital. |
| Scene 3 |  | I have ben practicing OB/GYN for the past 20 years, and I would like to spend a few minutes today discussing urgent maternal warning signs. If you are pregnant, or had a baby in the last two months, and experience any of these signs, you should call your doctor, or seek medical help at the nearest hospital. |
| Scene 4 |  | First, I would like to discuss signs of pre-eclampsia. Pre-eclampsia is when a woman develops high blood pressure because of her pregnancy. If pre-eclampsia is not treated, it can be life threatening. |
| Scene 5 |  | One sign of pre-eclampsia is a severe headache, that won’t go away with pain medication, or gets worse over time. There are other signs of pre-eclampsia. |
| Scene 6 |  | Some women will experience changes in their vision, such as blurry vision, flashes of light, or seeing spots. |
| Scene 7 |  | Other women that have pre-eclampsia may also have extreme swelling in their face or hands . . . |
| Scene 8 |  | . . . or have severe nausea or vomiting that starts long after morning sickness has ended. |
| Scene 9 |  | If you have any of these signs, your doctor can help determine if is pre-eclampsia or another problem. Most of the time, with early medical care, patients can recover from pre-eclampsia without long-term complications. |
| Scene 10 |  | There are other warning signs to look out for during pregnancy. |
| Scene 11 |  | If you have severe abdominal pain that won’t go away . . . |
| Scene 12 |  | . . . Or vaginal bleeding, these could be signs of the placenta beginning to separate too soon. Or that you are going to go into labor too soon. |
| Scene 13 |  | Also, if you feel like your baby has stopped moving, or is not moving as much as before, this could be a sign of a problem. |
| Scene 14 |  | After delivering your baby, it is normal to bleed for a few weeks. |
| Scene 15 |  | If the bleeding is very heavy, soaking a pad from front-to-back and side-to-side within one hour, or if there are large clots, the size of an egg, these could be signs that you are losing too much blood after birth. |
| Scene 16 |  | Pregnant and postpartum women are also at risk for developing blood clots. Swelling, or redness and pain in the leg, is a sign of a blood clot in the legs. |
| Scene 17 |  | Other signs of blood clots, or possible heart problems, include chest pain, which can feel like tightness in your chest, or a fast beating heart |
| Scene 18 |  | . . . Difficulty breathing and dizziness or fainting. |
| Scene 19 |  | If you develop a fever during pregnancy, or within a few weeks of having a baby, this could be a sign of an infection that needs treatment from your healthcare provider. |
| Scene 20 |  | Finally, many pregnant and postpartum women struggle with depression. Depression isn’t anyone’s fault, and it can be treated. |
| Scene 21 |  | If you have thoughts of hurting yourself or your baby, this is a medical emergency and you should seek immediate help. |
| Scene 22 |  | For more information on these signs, you can visit the Council on Patient Safety in Women’s Healthcare webpage on maternal warning signs, or talk to your healthcare provider. |
| Scene 23 |  | Please share these warning signs with your family, so that they can help you stay health. And remember, if you experience any of these signs, or you fel like something isn’t right and you’re worried about your health, contact your doctor right away. |
| Scene 24 |  | If you can’t reach your doctor, go to emergency room. And remember to say that you are pregnant or have ben pregnant recently. |
| Scene 25 |  | Our goal is to have a healthy mom, and a healthy baby. Thank you. |
| Scene 26 |  | [Exit music.] |
