# Supplementary material for: Maternal Warning Signs Education During Home Visiting: Results from a Formative Evaluation in Maryland
Source: Womens Health Rep (New Rochelle). 2022 Jul 11;3(1):633–42. doi: 10.1089/whr.2022.0027 (PMC9380880; doi:10.1089/whr.2022.0027)
Supplement: Supplemental data [file Suppl_TableS2.docx]

**Supplemental File 2. Description of common home visiting program models in Maryland**

| **Program Model** | **Key Characteristics of the Program Model** |
| --- | --- |
| Health Families America | - **Intended population:** “Parents facing challenges such as single parenthood; low income; childhood history of abuse and other adverse child experiences; and current or previous issues related to substance abuse, mental health issues, and/or domestic violence.” - **Enrollment/eligibility period:** Prenatally or within the first three months after the child’s birth. Eligibility ends when the child reaches the age of three to five, depending on site policies. - **Services provided:** “One-on-one home visits, monthly group meetings, developmental screenings, and linkages and connections for families to needed resources.” - **Frequency/number of home visits:** Weekly hour-long home visits until the child reaches six months of age, then less frequent. |
| Parents as Teachers | - **Intended population:** “Families with high-needs characteristics. Parents as Teachers affiliates select the specific characteristics and eligibility criteria of the target population they plan to serve. Such eligibility criteria might include children with special needs, families at risk for child abuse, low-income families, teen parents, first-time parents, immigrant families, low-literate families, parents with mental health or substance use issues, or families experiencing homelessness or unstable housing.” - **Enrollment/eligibility period:** Any time between pregnancy and the child reaching kindergarten; some sites may limit eligibility to families with children under 3. - **Services provided:** One-on-one home visits focused on “three areas of emphasis—parent-child interaction, development-centered parenting, and family well-being;” group connections; h**ealth, hearing, vision, and developmental screenings for children; linkages and connections for families to needed resources.** - **Frequency/number of home visits:** At least 12 hour-long home visits per year for at least two years. |
| Early Head Start (Home-Based Services Option) | - **Intended population:** Low-income pregnant women and families. - **Enrollment/Eligibility period:** From pregnancy until the child reaches age 3, depending on site policies. - **Services provided:** “Early, continuous, intensive, and comprehensive child development and family support services.” Includes “a minimum of one weekly 90-minute home visits and two group socialization activities per month for parents and their children.” - **Frequency/number of home visits:**  Minimum of weekly 90-minute home visits. |

Notes: ^1^Information sourced from program overviews from the U.S. Department of Health and Human Services webpage, “Home Visiting Evidence of Effectiveness,” accessed May 2022 (https://homvee.acf.hhs.gov/HRSA-Models-Eligible-MIECHV-Grantees); ^2^Information sourced from the Health Resources and Services Administration webpage, “Healthy Start,” accessed Mary 2022 (https://mchb.hrsa.gov/programs-impact/healthy-start).
